# Efficacy, safety, and controversy of ultrasound-guided radiofrequency ablation in the treatment of T1N0M0 papillary thyroid carcinoma

**DOI:** 10.3389/fonc.2022.1068210

**Published:** 2022-12-20

**Authors:** Zhang Yi, Li Siyu, Fu Lijun, Zhang Danhua, Li Jianhua, Qiu Xinguang

**Affiliations:** ^1^Department of Thyroid surgery, First Affiliated Hospital of Zhengzhou University, Zhengzhou, China; ^2^Physical Examination Center, First Affiliated Hospital of Zhengzhou University, Zhengzhou, China

**Keywords:** radiofrequency ablation, papillary thyroid carcinoma, ultrasound, lymph node metastasis, delayed surgery

## Abstract

**Objective:**

To evaluate the safety effect, and controversy on the treatment outcomes of radiofrequency ablation (RFA) for T1N0M0 papillary thyroid carcinoma (PTC).

**Materials and methods:**

This study is assessed the medical records of 142 patients with primary T1N0M0 PTC tumors after RFA between 2014 and 2022. 4 patients underwent delayed surgery (DS) after RFA and 411 T1N0M0 patients underwent DS were recorded. Outcomes were compared between RFA and DS groups after propensity score matching (PSM).

**Results:**

The maximal diameter (MD) and volume (V) increased in months 1 (P < 0.01) and reduced after the 6-month follow-up (all P < 0.01). The disappearance and disease progression rates were 53.5% and 2.1%, respectively. The complication and disease progression rates had no significant difference between RFA and DS (P>0.05). In some cases, the tumors were not fully inactivated after RFA, and the central compartment lymph node (CCLN) were metastasis. The CCLN metastasis rate was 13.4%. MD, V and clustered calcifications were independent risk factors for CCLN metastasis by univariate analysis.

**Conclusions:**

RFA is an effective and safe treatment option in selected patients with solitary T1N0M0 PTC. There are the risks of tumor incompletely ablated and CCLN metastasis.

## Introduction

Papillary thyroid carcinoma (PTC) is the most common thyroid malignancy with an ever-increasing yearly incidence rate (1, 2). With the development of high-frequency ultrasound (US) and biopsy techniques, more cases of papillary thyroid microcarcinoma (PTMC) are being identified (3, 4). Although there are many controversies regarding the treatment strategy of PTMC without lymph node metastasis (LNM), traditional or endoscopic thyroidectomy remains the primary treatment strategy (5).

The American Thyroid Association guidelines introduced active surveillance (AS), instead of surgery, due to the surgical complications and low risk of metastasis associated with PTC (6–8). Radiofrequency ablation (RFA) has been rapidly promoted as the first-line approach for treating benign thyroid tumors or as a palliative treatment for metastatic lymph nodes in patients with thyroid cancer. RFA provides a new option for these patients because it is minimally invasive, has no impact on aesthetic appearance, and results in less trauma and scanty complications for the patient.

RFA was conceived initially as a way to treat benign thyroid nodules (BTNs) (9, 10) and is more frequently used in treating primary thyroid malignancies (11–13). In 2017, the Korean Radiofrequency Ablation Association published guidelines that proposed thermal ablation as a treatment method for thyroid cancer with LNM and PTMC and provided a basis for using RFA in PTMC (14). The Chinese Medical Doctors’ Association and European Thyroid Association successively produced guidelines for the thermal ablation of thyroid tumors (15, 16). Many studies have confirmed that RFA is effective and safe for treating BTNs (17–19) which the incidence of RFA-associated complications, such as dysphonia, hypocalcemia and bleeding, is very low (20–22). Recent several studies have shown that RFA is effective for treating PTMC (23–25). However, studies on the prognosis, safety, and efficacy of RFA in treating T1N0M0 PTC are still insufficient. In addition, current studies mostly explore the efficacy and safety of thermal ablation. However, some potential risks are disregarded, including inadequate tumor inactivation, missed central compartment lymph node (CCLN) metastasis.

Therefore, this study aimed to explore the prognosis, safety, and efficacy of RFA in treating T1N0M0 PTC moreover to analyze risk factors of postoperative recurrence. Some cases underwent delayed surgery (DS) after RFA to evaluate tumor inactivation. Thus, the patients with T1N0M0 disease which is performed DS were selected to assess the proportion and related factors of missed CCLN metastasis. This was done as the results might provide a more impartial evaluation of RFA in patients with T1N0M0 PTC possible can be benefit to patients when they select between RFA and DS.

## Materials and methods

This retrospective study was approved by the ethics committee at our respective institutes (Reference No. 2022-KY-0844-001) and the requirement for informed consent was waived. Informed consent was obtained from all patients for the treatment delivered.

The inclusion criteria were (a) maximum diameter (MD) less than 20 mm, (b) puncture biopsy indicating PTC with no invasion and rupture of the thyroid capsule on the preoperative US, (c) no invasion of surrounding tissues or regional lymph nodes, or distant metastasis, (d) ineligibility for or refusal to undergo surgery, and (e) at least 6-month duration of follow-up. Exclusion criteria were (a) multiple PTCs, (b) tumor located in the isthmus of the thyroid, (c) age younger than 18 years or pregnancy, and (d) unavailability of complete follow-up data.

Pre- and postoperative US examinations were performed by expert doctors (more than 5 years of experience) at our hospital’s Ultrasound Department. US imaging provided data on tumor location, size (three meridians), and US characteristics of the tumor. The volume was calculated using:


V=πABC/6

(where V is the volume, A is the MD, and B and C are the other two vertical diameters). The pathology results of preoperative fine-needle aspiration (FNA) indicated PTC presence, while *BRAF^V600E^* gene detection was used for auxiliary judgment when necessary. Computed tomography imaging of the neck and chest was performed to detect lymph node and distant metastasis.

The VIVA RF Generator (STARmed, Gyeonggi-do, South Korea) was used in this study. An 18-gauge, modified, monopolar, and internally cooled RFA antenna with a 1-cm active tip and a 7-cm shaft length was used; this was specifically modified for the ablation of thyroid nodules. The patients were asked to remain the supine position, and the shoulder and neck were padded and hyperextended to expose the neck fully. Routine disinfection and towel laying were performed. Lidocaine (2%) was used as the local anesthetic. Under US guidance, an electrode was inserted along the local anesthesia track into the nodule. Fixed ablation was used for small nodules, and multipoint or mobile ablation (moving-shot technique) was used for large nodules with multiple expected movements of the applicator into the target thyroid nodule. The treatment was considered complete when the strong echo range exceeded the original tumor margin by 5 mm (minimum 2 mm). The liquid isolated the recurrent laryngeal nerve (RLN), internal jugular vein, and common carotid artery. The average ablation time was 4.15 ± 1.48min, and compression was applied for 20–30 min postoperatively. Elevations of blood pressure, dysphonia, bleeding, and other complications were monitored.

All patients returned for follow-up US examinations at 1, 3, 6, and 12 months postoperatively, and every 6 months after that. Follow-up data were mainly based on thyroid US. The important indicators were as follows: 1. the size and volume of the lesion, and the percentage of reduction in the volume of the lesion after RFA, 2. the presence of recurrence and metastasis based on puncture biopsy results, when necessary, 3. the scope and morphology of necrosis in the ablation area, and 4.tumor disappearance and complication rate (the standard for image-guided thyroid ablation) (26).

Data analysis was performed using the SPSS software (SPSS for windows 21.0, SPSS, Chicago, IL). Descriptive statistics that are normally distributed are expressed as mean ± standard deviation, and categorical variables are given as frequency and percentage. Propensity score matching (PSM) was performed using the Stata 15.The paired t-test was used to assess differences between pretreatment and posttreatment, and the chi-squared test was used for comparing groups. Univariate analysis and log-rank test were separately used to analyze the factors of CCLN metastasis not identified on US. A value of P < 0.05 was considered to define statistically significant differences.

## Results

### Patient demographic and tumor characteristics

**Figure 1:**
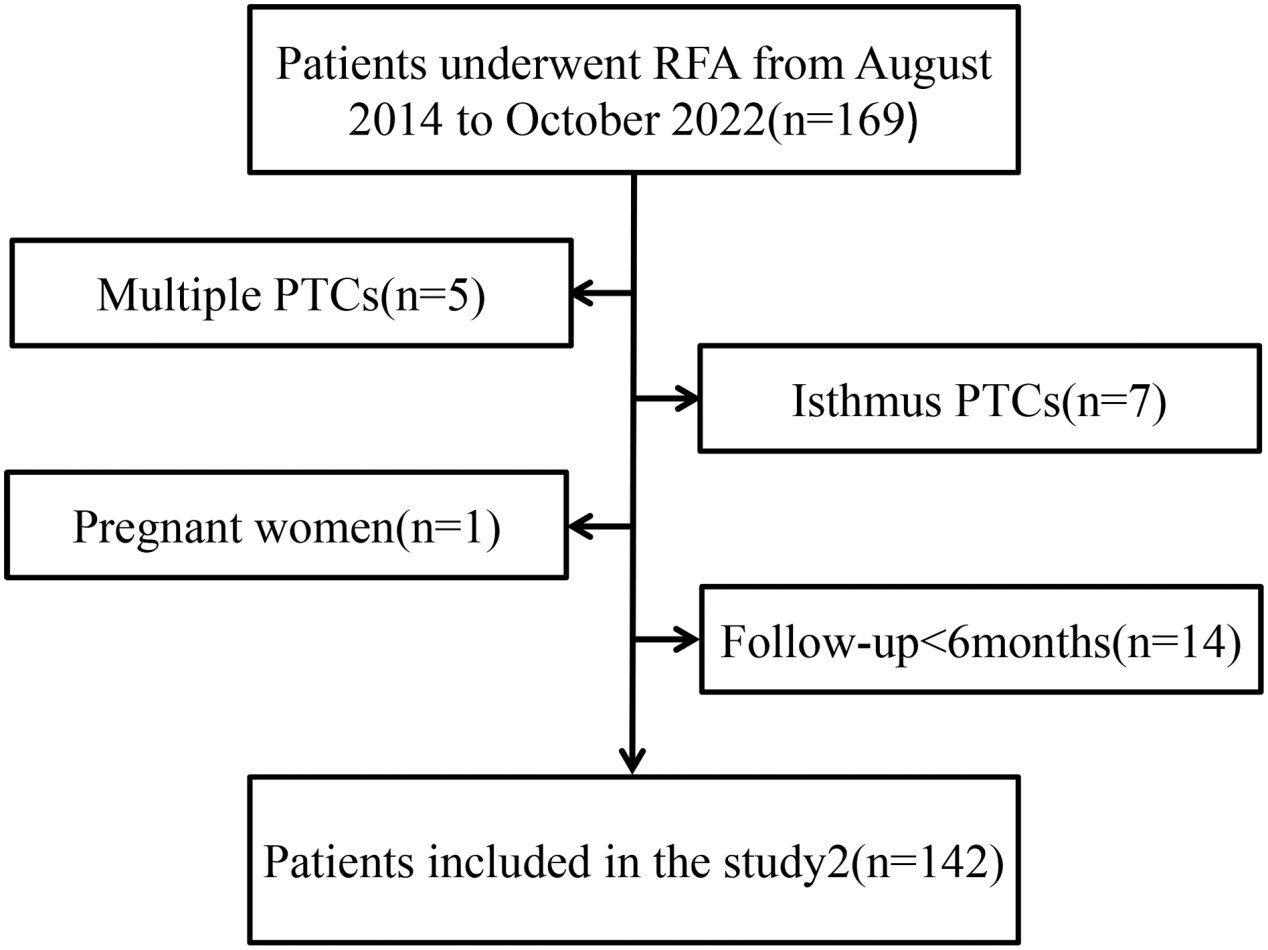
Research flowchart. RFA, radiofrequerncy, ablation, PTC, papillary thyroid carcinoma.

Since August 2014 to October 2022, the total numbers of patients (142) which is included 36 males 106 females respectively, aged 19–81 (mean age, 46.40 ± 14.30 years) underwent US-guided FNA or core-needle biopsy (CNB), and the pathology results were PTC (**Figure 1**). T1N0M0 PTC refers to a single thyroid nodule with a diameter of less than 2 cm, clear pathological indication, and no LNM. These 142 patients further underwent US-guided RFA. In addition, 411 patients who underwent DS and prophylactic CCLN dissection and whose preoperative US showed T1N0M0 PTC in the same period were selected. 142 cases were selected to compare after propensity score matching (PSM) (**Table 1**). Some exceptional cases were collected, including 4 patients who underwent DS after RFA.

**Table 1:** Demographic characteristics of the study population.

Variable	Before PSM		P Value	After PSM		P Value
	RFA(n=142)	DS(n=411)		RFA(n=142)	DS(n=142)	
Age (year)*	46.40 ± 14.30	50.06 ± 9.59	0.005	46.40 ± 14.30	48.84 ± 8.74	0.084
Sex						
Female	106 (74.6)	327 (79.6)	0.221	106 (74.6)	109(76.8)	0.678
Male	36 (25.4)	84 (20.4)		36 (25.4)	33 (23.2)	
Location						
Left/Right	63/79	191/220	0.664	63/79	66/76	0.721
upper/middle/lower	41/46/55	119/103/189	0.186	41/46/55	33/42/67	0.328
deep/middle/shallow	42/71/29	131/211/69	0.606	42/71/29	46/75/21	0.456
Clustered calcifications(-/+)	40/102	149/262	0.080	40/102	41/101	0.895
Hypoechoic(-/+)	10/132	32/379	0.773	10/132	13/129	0.514
Diameter (mm)*	6.37 ± 3.14	7.73 ± 3.48	<0.001	6.37 ± 3.14	6.92 ± 2.91	0.124
Volume (mm^3^)*	156.65 ± 251.26	755.82 ± 1 424.97	<0.001	156.65 ± 251.26	194.81 ± 291.35	0.216
Thyroid function						
FT3 (pmol/L)	4.89 ± 0.87	4.83 ± 1.01	0.489	4.89 ± 0.87	4.79 ± 1.02	0.362
FT4 (pmol/L)	12.80 ± 2.90	13.06 ± 3.08	0.380	12.80 ± 2.90	12.71 ± 3.21	0,795
TSH (uIU/ml)	3.31 ± 1.57	3.28 ± 1.57	0.855	3.31 ± 1.57	3.42 ± 1.53	0.547
Time of diagnosis	18.17 ± 14.57	24.60 ± 15.78	<0.001	18.17 ± 14.57	20.75 ± 14.10	0.131

Unless otherwise specified, data are the number of patients. * Data presented as Means ± standard deviation. PSM, propensity score matching. Normal range: FT3 3.28~6.47 pmol/L, FT4 7.90~18.40 pmol/L, TSH 0.56~5.91 uIU/ml.

### The curative effects

Postoperative contrast-enhanced (CE) US imaging was performed to ensure that all tumors were completely enhanced by different degrees at the end of ablation. The filling defect at the ablation site of the CE US was an anechoic area, suggesting that the lesion was completely ablated. The ablation range was 2-3 mm larger than the lesion range. The ablation power and range were appropriately reduced at the upper and lower poles, especially for the lesions near important tissues such as RLN, esophagus, blood vessel etc.

**Figure 2:**
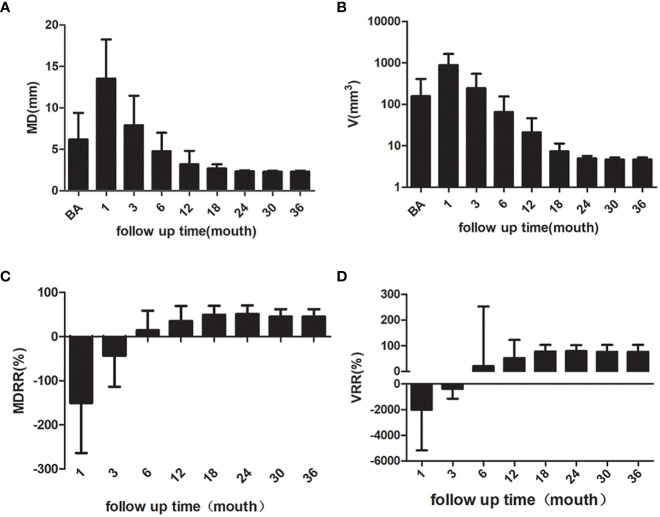
The MD, V, MDRR and VRR of the nodules. **(A)** MD of the nodules **(B)** V of the nodules **(C)** MDRR of the nodules **(D)** VRR of the nodules. MD, maximum diameter; V, volume; MDRR, maximal diameter reduction ratio; VRR, volume reduction ratio.

The mean MD of the nodules before ablation was 6.37 ± 3.14 mm, and the mean V was 156.65 ± 251.26 mm^3^. The MD and V of the ablation low echo area were larger than those of the original tumor at the evaluations performed during months 1 after ablation (P < 0.001); this is due to the ablation range having been 2-3 mm larger than the lesion range. The MD and V of the ablation low echo area were less than those of the original tumor at the 6-month and longer follow-up examinations after ablation (P < 0.001 for all). The changes are shown in **Table 2**. The MD and V were significantly reduced at the 6–24-month follow-up, while the reduction was minimal after 24 months, which has shown in **Figure 2**. The tumor disappearance rate was 53.5% (76/142).

**Table 2:** The changes of mean maximal diameter and volume after ablation over 1 year.

Follow up time	MD (mm)	*P* Value	Volume (mm^3^)	*P* Value
Preablation (n=142)	6.37 ± 3.14		156.63 ± 222.69	
Postablation				
1 month (n=142)	11.15 ± 4.78	<0.001	586.79 ± 653.12	<0.001
3 month (n=142)	6.90 ± 3.21	0.031	180.43 ± 246.94	0.173
6 month (n=117)	4.73 ± 1.96	<0.001	58.01 ± 78.22	<0.001
12 month (n=50)	4.13 ± 1.36	<0.001	31.53 ± 35.73	<0.001

Data are means ± standard deviation. P value is preablation vs postablation respectively. MD, maximum diameter.

The American Joint Committee classifies the T1 PTC into T1a (tumor ≤ 1 cm) and T1b (tumor > 1 cm but ≤ 2 cm). The follow-up data for T1a and T1b PTC are shown in **Table 3**. The MD and V of T1a of the ablation zone at the 12-month follow-up were less than those for the original tumor (P < 0.001 for both). The changes of MD and V in T1b PTC at the 12-month follow-up were significant (P = 0.003 and P = 0.004, respectively).

**Table 3:** The comparison of T1a and T1b PTC.

Variable	T1a PTC (n=122)	T1b PTC (n=20)	P
Preablation			
MD (mm)	5.37 ± 1.96	12.39 ± 2.11	< 0.001
Volume (mm^3^)	79.89 ± 82.04	624.75 ± 238.05	< 0.001
Postablation			
MD (mm)			
6 month	4.27 ± 1.62	6.98 ± 1.96	< 0.001
12 month	3.81 ± 1.23	5.06 ± 1.34	0.003
Volume (mm^3^)			
6 month	39.78 ± 52.31	146.40 ± 116.68	< 0.001
12 month	23.39 ± 22.35	55.30 ± 54.39	0.004
Disappearance rate*	72 (59%)	4 (20%)	0.001
Disease progression*	2 (1.6%)	1 (5%)	0.368

Data are means ± standard deviation. * Data presented as number.

PTC, papillary thyroid carcinoma; MD, maximum diameter.

### The safety and treatment efficacy

The complication rate at the most recent follow-up was 2.8% (4/142), with hematoma and dysphonia. There was no significant difference in the complication rate between RFA and DS in the short term (P = 0.518, **Table 4**). The disease progression rate after RFA was 2.1% (3/142) in the present study. The disease progression rate after DS was 1.4% (2/142). There was no significant difference in disease progression rate between RFA and DS in the short term (P =0.652, **Table 4**).

**Table 4:** The complications and disease progression of RFA and DS.

Variable	RFA (n=142)	DS(n=142)	P
Complication	4	6	0.518
Hematoma	1	1	
Hypocalcemia	0	1	
Dysphonia	3	4	
Disease progression	3	2	0.652
New tumors	1	1	
LNMs	2	1	

Data are expressed as number of findings. RFA, radiofrequency ablation.

DS, delayed surgery; LNM, lymph node metastasis.

**Figure 3:**
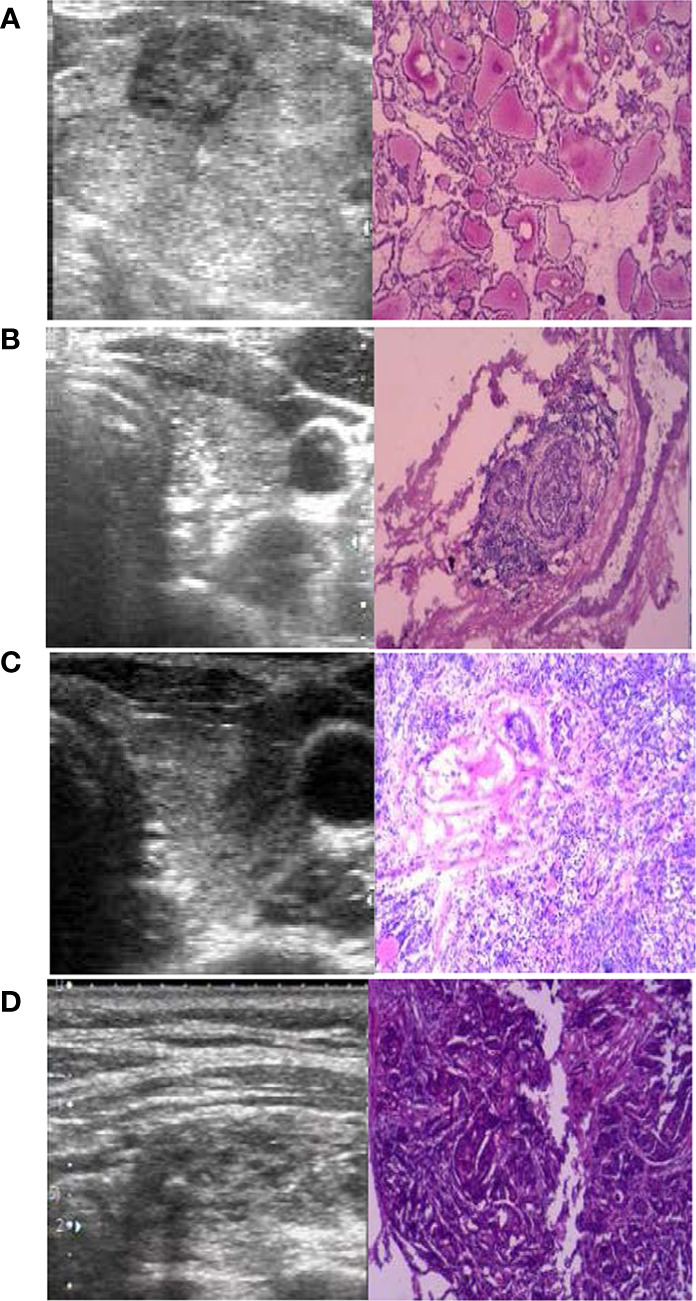
The preoperative ultrasound (US) and postoperative pathology (×100). **(A)** The ablation region had a new PTC four years after radiofrequency ablation (RFA). **(B)** The lymph nodes were metastasis (positive/all was 7/9) 3 months after RFA. **(C)** The PTC was not completely ablated 3 months after RFA. **(D)** The PTC was completely ablated 1 months after RFA.

The four cases that underwent DS after RFA were assessed to understand whether the tumor was completely ablated. All RFA were performed by doctors who had more than 100 procedures. The first patient underwent DS due to enlargement of thyroid nodules 4 years after RFA; the postoperative pathology showed that the ablation region had a new tumor (PTC, MD was 3 mm). The second patient underwent DS because CCLN metastasis was observed 3 months after RFA; the postoperative pathology showed the tumor was not ablated, and there was CCLN metastasis (metastasis/all was 7/9). The remaining 2 patients underwent concomitant RFA and FNA followed by DS as the puncture biopsy results indicated PTC. The pathology suggested that the tumor was completely ablated in one case but not in the other. The US and pathology images are shown in **Figure 3**.

To calculate the characteristics of CCLN metastasis in T1N0M0 PTC, the data of 142 patients were analyzed who underwent DS and CCLN dissection. The postoperative pathology revealed that the rate of LNM was 13.4% (19/142). The results of the chi-squared test and t-test showed that the independent risk factors for CCLN metastasis were MD, V and clustered calcifications as shown in **Table 5**.

**Table 5:** Univariate analysis of CCLN metastasis in T1N0M0 PTC.

Parameters	CCLN metastasis (+) (n=19)	CCLN metastasis (-) (n=123)	*P*
Age(year)*	46.79 ± 9.69	49.15 ± 8.58	0.274
Sex(Female/Male)	15/4	94/29	0.807
MD (mm)*	9.58 ± 3.58	6.51 ± 2.57	0.002
Volume (mm^3^)*	422.71 ± 322.27	159.61 ± 271.02	0.003
Location			
right/lift	13/6	60/63	0.218
upper/middle/lower	6/6/7	27/36/60	0.560
deep/middle/shallow	2/8/9	19/38/66	0.660
Clustered calcifications(-/+)	7/12	96/27	<0.001

Data are the number of patients. *Data presented as Means ± standard deviation and use t test. CCLN, central compartment lymph node; MD, maximum diameter.

## Discussion

There is still no clear consensus on the optimal management strategy for PTC. The controversy surrounding management approaches includes the choice among open surgery, thermal ablation, and AS. RFA has been rapidly promoted as the first approach for treating benign thyroid tumors or even for the palliative treatment of metastatic lymph nodes in patients with thyroid cancer because it is minimally invasive and has no impact on aesthetic appearance (27). Multiple studies have reported that using RFA for patients with PTMC was safe, effective, and reliable (28, 29). Our study confirmed the safety, efficacy, and reliability of RFA in treating T1N0M0 PTC.

In this study, the tumor disappearance, disease progression, and complication rates of tumors after RFA were 53.5%, 2.1% and 2.8%, respectively. Moreover, our study showed that the tumor disappearance rate of T1N0M0 PTC was lower than the results of a meta-analysis of data from 12 studies (combined n = 1187) that showed the complete disappearance rate of PTMC was 76.2% (30). The reason may be it is required greater range and power when RFA in treat of T1N0M0 PTC. The tumors showed reduction in 12 month both at T1a and T1b groups. RFA is a feasible and effective treatment option in selected patients with T1a and T1b PTC.

The complication rate of this study is similar to result which has shown in studies of PTMC and BTNs. In the terms of RFA complications, dysphonia was the most common and occurred secondary to recurrent thermal injury of the RLN. As RLN edema subsided, the dysphonia was resolved within 6 months. In some previous studies, the dysphonia incidence rate due to DS was 3-4% (31–33), which was insignificantly different to the result in this study (2.8%). Wei et al. (34) reported that there was no difference regarding disease progression and complications between microwave ablation and DS. In this study, the complication rates following RFA were the same as DS (P = 0.518). There were no differences in disease progression between RFA and DS (P = 0.652). To reduce the incidence of thermal injury of the RLN, the surgeon should employ hydro-dissection and accurate puncturing, as well as the ablation time and subsequent release of energy should be reduced. This study observed that the safety and efficacy of RFA in T1N0M0 PTC were favorable when compared with DS.

During the 24-month follow-up examination, we found that RFA was reliable regarding the tumor gradually reduced or disappeared. This study showed favorable results in the medium- and short-term follow-up examinations. Although PTC progresses slowly, several controversial issues cannot be ignored until the long-term follow-up examination results are available or until further research is conducted. However, while there are several studies on the effectiveness of RFA, only a few investigate its limitations, which may mislead doctors or patients into overconfidence about RFA. The most controversial issues of RFA are whether the tumor is entirely necrotic and LNM. Four cases that underwent DS after RFA were analyzed to investigate tumor necrosis, and the results proved that tumor is not removed completely and LNM existed. Sun et al. (35) reported that of 21 patients who underwent surgery after RFA, 33.3% had bilateral cancer and 47.6% had CCLN; similar results (66.7%, 8/12) were reported by Ma et al. (36).Due to the lack of cases, the extent of these situations is unknown.

To explore LNM, we collected clinical data from T1N0M0 PTC patients who had performed DS and CCLN dissection, the same inclusion criteria as RFA. The requirement of RFA make the inclusion criteria different from traditional T1N0M0 PTC. The tumors should be single and located in some distance from the boundary of envelope, trachea, esophagus, recurrent laryngeal nerve. The patients should have no family history, no radiation history. After using PSM to reduce the dimension of the data, the CCLN metastasis rate was 13.4% which significantly lower than traditional T1N0M0 PTC (37, 38). The univariate analysis showed that MD, V, and clustered calcifications correlated significantly with CCLN metastasis. Compared with hypoechoic nodules, clustered calcifications were strongly correlated with CCLN metastasis. In some studies, CCLN metastasis was associated with PTC recurrence rates (39–41). As the risk for recurrence of LNM remains, our study found that patients with smaller-diameter and no cluster calcificationare are more suitable for RFA.

This study found that appropriately expanding the scope of operation, prolonging the operation time, and performing postoperative CE US to ensure complete ablation of lesions can prevent recurrence. RFA should be performed from top to bottom and deep to shallow regions to ensure no omissions. Few patients with CCLN metastasis bear a high risk of recurrence, often undergoing DS after RFA. Doctors should adhere strictly to the indications of RFA and fully inform patients when discussing RFA as an option with PTC patients, as undergoing DS after RFA increases the financial burden of patients and may lead to disease progression. A significant advantage was that DS performed after RFA was reasonably straightforward based on clinical experience; only slight tissue adhesions were found intraoperatively. In addition, our study found that most patients receiving minimally invasive treatments were females aged 18-30 years and patients with jobs that required a high aesthetic appearance. These patients refused to undergo open surgery; thus, RFA may be the appropriate alternative treatment. Open surgery should be considered the primary treatment option for patients who prefer radical treatment over aesthetic appearance and minimally invasive surgery.

This study confirmed the safety and reliability of RFA in treating T1N0M0 PTC. Nonetheless, patient subsets that can and cannot undergo RFA are still not known. Further studies are required to fully predict which groups of patients with thyroid cancer are suitable for RFA and to detect LNM that cannot be detected by color Doppler ultrasound. Furthermore, future studies should answer whether RFA is better than AS and whether it provides the same results and outlook of open surgery, as well as determine which options are available for any remaining residual tumor post-RFA.

Our research had some limitations. First, this was a single-center study. Second, the mean follow-up period in this study was short based on the progression of PTC. Third, this study lacks data regarding the pathology diagnosis of RFA. Lastly, the CCLN metastasis ratio was inferred by pathological results obtained during DS, rather than after RFA.

## Conclusions

In conclusion, RFA is a safe and effective treatment for T1N0M0 PTC but is associated with a higher recurrence risk than DS. We found that RFA was associated with fewer postoperative complications and less trauma to the patients, had no impact on aesthetic appearance, and required only local anesthesia. Moreover, RFA is more suitable for females aged 18-30 years, elderly patients or patients with serious other illnesses. However, the incomplete tumor inactivation and LNM make RFA unsuitable to replace open surgery as the primary treatment option. Therefore, the indications of RFA should be carefully considered, and the patients should be fully informed.

## Data availability statement

The original contributions presented in the study are included in the article/supplementary material. Further inquiries can be directed to the corresponding author.

## Ethics statement

The studies involving human participants were reviewed and approved by the ethical and scientific review board of the First Affiliated Hospital of Zhengzhou University. Written informed consent for participation was not required for this study in accordance with the national legislation and the institutional requirements. Written informed consent was obtained from the individual(s) for the publication of any potentially identifiable images or data included in this article.

## Author contributions

ZY, LS, FL, ZD, LJ: Study design and manuscript writing. ZY, LS, FL: Studies selecting and data analysis. ZY, ZD, LJ, QX: Study quality evaluating. ZY, QX: Manuscript revising. All authors have read and approved the final manuscript. All authors contributed to the article and approved the submitted version.
